# Deletion Genotypes Reduce Occlusion Body Potency but Increase Occlusion Body Production in a Colombian *Spodoptera frugiperda* Nucleopolyhedrovirus Population

**DOI:** 10.1371/journal.pone.0077271

**Published:** 2013-10-08

**Authors:** Gloria Barrera, Trevor Williams, Laura Villamizar, Primitivo Caballero, Oihane Simón

**Affiliations:** 1 Instituto de Agrobiotecnología, CSIC-Gobierno de Navarra, Mutilva Baja, Navarra, Spain; 2 Corporación *Colombiana* de Investigación Agropecuaria (CORPOICA), Bogotá, Colombia; 3 Instituto de Ecología AC, Xalapa, Veracruz, Mexico; 4 Departamento de Producción Agraria, Universidad Pública de Navarra, Pamplona, Navarra, Spain; Wuhan Bioengineering Institute, China

## Abstract

A Colombian field isolate (SfCOL-wt) of *Spodoptera frugiperda* multiple nucleopolyhedrovirus (SfMNPV) is a mixture of different genotypes. To evaluate the insecticidal properties of the different genotypic variants, 83 plaque purified virus were characterized. Ten distinct genotypes were identified (named A through J). SfCOL-A was the most prevalent (71±2%; mean ± SE) showing a *Pst*I restriction profile indistinguishable to that of SfCOL-wt. The remaining nine genotypes presented genomic deletions of 3.8 - 21.8 Kb located mainly between nucleotides 11,436 and 33,883 in the reference genome SfMNPV-B, affecting the region between open reading frames (ORFs) *sf20* and *sf33*. The insecticidal activity of each genotype from SfCOL-wt and several mixtures of genotypes was compared to that of SfCOL-wt. The potency of SfCOL-A occlusion bodies (OBs) was 4.4-fold higher than SfCOL-wt OBs, whereas the speed of kill of SfCOL-A was similar to that of SfCOL-wt. Deletion genotype OBs were similarly or less potent than SfCOL-wt but six deletion genotypes were faster killing than SfCOL-wt. The potency of genotype mixtures co-occluded within OBs were consistently reduced in two-genotype mixtures involving equal proportions of SfCOL-A and one of three deletion genotypes (SfCOL-C, -D or -F). Speed of kill and OB production were improved only when the certain genotype mixtures were co-occluded, although OB production was higher in the SfCOL-wt isolate than in any of the component genotypes, or mixtures thereof. Deleted genotypes reduced OB potency but increased OB production of the SfCOL-wt population, which is structured to maximize the production of OBs in each infected host.

## Introduction

Viruses in the family Baculoviridae are characterized by a high intraspecific heterogeneity in isolates from distant geographic regions (interpopulation diversity) [1,2] and also within single isolates that can exist as mixtures of genotypes (intrapopulation diversity) [3-7]. Genotypic variants can be the result of recombination during simultaneous infections by multiples genotypes or can be due to genetic drift by mutation during replication [8,9]. Moreover, genomic variability has important effects on baculovirus fitness parameters related to the pathogenicity, speed of kill and production of viral occlusion bodies (OBs) [7,8].

Genotypic variability of the *Spodoptera frugiperda* multiple nucleopolyhedrovirus (SfMNPV) has been determined by characterization of different geographical isolates [1,2,10] and by isolating the genotypic variants present in particular field isolates using *in vitro* techniques [7,11]. In a Nicaraguan field isolate (SfNIC) the OBs of all genotypic variants were less pathogenic, as determined by concentration-mortality metrics, than the wild type OBs or those of experimental mixtures of genotypes [7,12,13]. In contrast, in a commercial isolate of *S. exigua* multiple NPV (SeMNPV), deletion genotypes reduced the activity of the wild-type population [14,15]. These findings have emphasized the need to evaluate interactions between genotypes within wild-type nucleopolyhedrovirus populations that can be highly advantageous during the process of selecting the active material for the development of baculovirus-based insecticides.

Previous studies on SfMNPV as a potential biological control agent in Colombia identified the SfCOL isolate as the most pathogenic of a total of 38 Colombian field isolates and the Nicaraguan isolate SfNIC previously characterized [10]. The objectives of the present study were to determine the genotypic diversity present in the SfCOL isolate and evaluate the contribution of the genotypic variants to the insecticidal properties of the natural isolate by examining key phenotypic traits, such as pathogenicity, virulence and OB productivity, of single genotypes alone and in mixtures with the dominant genotype.

## Materials and Methods

### Insects source and rearing

Larvae of *S. frugiperda* were obtained from a laboratory colony established in the Biological Control Laboratory of the Colombian Corporation of Agricultural Research (Corpoica) using larvae collected from experimental maize crops located in Corpoica Research Center "La Libertad" (Villavicencio, Colombia). Specific permission requirements were not necessary to collect larvae as this location is not in a protected area and *S. frugiperda* is not a protected species. This insect colony was periodically refreshed with field-collected insects and maintained at 25±1 °C, 75±5% RH (relative humidity) and 16 h light: 8 h dark photoperiod on a wheatgerm-based semisynthetic diet [16]. However, during rearing this colony collapsed and a second colony was established shortly after that with larvae collected from maize crops at the same site. 

### Virus isolate and amplification

The Colombian isolate SfCOL [10] was amplified in *S. frugiperda* larvae. OBs from cadavers were purified [17], and resuspended in milli-Q water. OB concentrations were determined using an improved Neubauer hemocytometer (Hawksley Ltd., Lancing, UK) under phase contrast microscopy at x400. Purified OBs were stored at 4 °C. 

### In vitro virus cloning

For the isolation of individual genotypes, SfCOL infected larvae were surface decontaminated with 70% ethanol. Hemolymph was taken by bleeding at 48 h post infection (h.p.i.) and diluted in sterile phosphate buffered saline (PBS). Sf9 cells were prepared at 2 x 10^6^ cells/well in 6-well tissue culture plates and incubated at 27 °C for 3 h, with TC100 medium supplemented with 1% penicillin/streptomycin (Gibco). The medium was then removed and 0.1 ml of serial 10-fold dilutions (from 10^-2^ to 10^-6^) of hemolymph were inoculated onto cells. After 1 h, the inoculum was removed and 3 ml of TC100 medium supplemented with 5% fetal calf serum (FSC, Gibco), 3% (w/v) SeaPlaque agarose and antibiotics were added to each well. Agarose was overlaid with 3 ml TC100 medium supplemented with antibiotics. The overlaid liquid was replaced every day. After 10 days, 248 well isolated plaques were picked individually using a sterile Pasteur pipette and transferred to a vial containing 0.1 ml of PBS. Volumes of 5 μl of this suspension were injected into *S. frugiperda* fourth instars for viral amplification. Virions were injected into larvae in order to avoid the potential loss of genotypes that were defective in genes necessary for oral infectivity, such as the *per os* infection factors (*pifs*). 

### Purification of OBs, DNA extraction and restriction endonuclease analysis

OBs were purified from dead diseased larvae by titration and centrifugation [17] and quantified by counting three times in a Neubauer hemocytometer. For DNA extraction, virions were released from OBs by mixing 100 μl of purified OB suspension at 1 x 10^9^ OBs/ml with 100 μl of 0.5 M Na_2_CO_3_, 50 μl of 10% sodium dodecyl sulphate (SDS) in a final volume of 500 μl and incubating for 10 min at 60 °C. Undissolved OBs were removed by low-speed centrifugation (3,800 × *g*, 5 min). Supernatant containing the virions was treated with 25 μl of proteinase K (20 mg/ml) for 15 min at 50 °C. Viral DNA was extracted using saturated phenol-chloroform followed by alcohol precipitation. The resulting pellet was resuspended in 50 to 100 μl of 0.1×TE (Tris-EDTA) for 10 min at 60 °C. DNA concentration was estimated using a UV-spectrophotometer at 260 nm (ND1000 - Thermo Scientific).

For restriction endonuclease analysis, samples were compared with the SfCOL-wt profile [10]. A sample of 2 μg of viral DNA was mixed with 10 U of one of the following restriction enzymes: *Pst*I, *Bam*HI or *Hin*dIII (Takara), and incubated for 4 to 12 h at 37°C. Reactions were stopped by mixing with 4 μl of loading buffer solution (0.25% w/v bromophenol blue, 40% w/v sucrose). Electrophoresis was performed using horizontal 1% agarose gels in TAE buffer (0.04 M Tris-acetate, 0.001 M EDTA, pH 8.0) at 20 V for 10 to 24 h. DNA fragments were stained with ethidium bromide and visualized on a UV transilluminator (Chemi-Doc, BioRad, California, USA). Genotypes that differed in one or more restriction profiles were each assigned a letter from the alphabet (SfCOL-A, -B, etc.).

### Construction of physical maps

Physical maps of the purified genotypes were constructed by comparison with the physical maps of SfCOL [10]. For this, restriction fragments obtained after treatment with different enzymes were compared with those of SfCOL, and co-migrating as well as genotype-specific fragments were identified. Based on this, preliminary physical maps were constructed. Two differential SfCOL genotype-specific *Pst*I fragments that were found to be characteristic of the *Pst*I profiles of SfCOL-C and -D genotypes, namely *Pst*I-E' fragment (7.5 Kb) and *Pst*I-C' fragment (9.1 Kb), respectively, were each cloned into pUC19 plasmid. The ligation reaction was prepared by mixing 1 µl (50 ng/µl) of purified vector pUC19, 5 µl of 10x ligase buffer, 1 µl (2U/µl) of ligase enzyme and 15 µl of the corresponding *Pst*I fragment obtained from excised pieces of agarose, incubated at 16 °C overnight and used to transform *Escherichia coli* cells (Invitrogen ElectroMAX DH10B-T1). The cells were plated on LB-ampicillin (100 mg/ml) plates containing IPTG (0.1 mM) and X-gal (40 µg/ml). White colonies that included the DNA fragment were amplified in liquid LB containing ampicillin (100 mg/ml) at 37 °C overnight. DNA was then extracted by alkaline lysis, digested with the relevant endonuclease and separated on 1% agarose and compared with total DNA digested from the SfCOL isolate. 

The cloned fragments were sequenced by primer walking using universal M13 and M13 reverse primers (Sistemas Genómicos, Paterna, Valencia, Spain). Sequence information was analyzed for the presence of open reading frames (ORFs) using Open Reading Frame Finder (NCBI). Homology searches were performed at the nucleotide and amino acid levels using all putative ORFs. DNA and protein comparisons with entries in the updated GenBank/EMBL, SWISS PROT and PIR databases were performed using BLAST (NCBI). Additionally, to confirm the deleted ORFs in SfCOL genotypes, some ORF regions were amplified by PCR using specific primers for different ORFs based on sequence from SfCOL [10] and SfMNPV-B genomes [18] (Table S1). These amplifications were compared with those obtained from SfCOL-wt, to confirm the deletions within these regions.

### Relative proportion of complete SfCOL-A genotype in the wild-type population

Once the physical maps were constructed and the gene content of each genotype had been determined, specific primers were designed in a region common to all genotypes and in a specific region of the complete SfCOL-A genotype in order to estimate the relative proportion of the complete genotype in the wild-type population by qPCR. A set of specific primers were designed that amplified in the *DNA polymerase* gene, common to all genotypes, and in the *egt* gene, present only in SfCOL-A (Table S1) using sequence information from the SfMNPV-B genome [18]. Non-template controls were analyzed for each set of primers designed in order to verify the absence of non-specific background signal. 

SfCOL wild-type DNA was extracted from OBs as previously described. Five different DNA extractions were performed in each of three replicates. All DNA measures were performed twice. The frequency of SfCOL-A was obtained by calculating the average of the fifteen samples (mean ± standard error). DNA concentration was quantified by spectrophotometry and by agarose gel electrophoresis, and diluted to 0.01 ng/μl. All reactions were performed using SYBR Green fluorescence in an ABI PRISM 7900HT Sequence Detection System (Applied Biosystems). The reaction mixture (10 µl) contained 5 µL SYBR Premix Ex *Taq* (2x), 0.2 µl of ROX Reference Dye (50x), 0.1 µl of each SfMNPV primer (10 pmol/µl) (Table S1) and 1 µl of DNA template. qPCR was performed under the following conditions: 95 °C for 30 s, followed by 45 elongation cycles of 95 °C for 5 s and 60 °C for 30 s and finally a dissociation stage of 95 °C for 15 s, 60 °C for 15 s and 95 °C for 15 s. Data acquisition and analysis were handled by Sequence Detector Version 2.2.2. software (Applied Biosystems). Known dilutions of SfCOL-A CsCl-purified DNA (10^-7^ - 10^-1^ ng/µl) were used as internal standards for each qPCR reaction. Melting-curve analysis was performed to confirm specific replicon formation in qPCR. 

### Production of OB mixtures and co-occluded mixtures of genotypes

Experimental mixtures were produced for the combinations of genotypes SfCOL-(A)+(C), -(A)+(D) and -(A)+(F) as previously described [12,19]. Briefly, the purified OB suspensions of each genotype were quantified by counting and adjusted to the same concentration. OBs of each genotype were then mixed in equal proportions and used as peroral inocula in insect bioassays described below. 

To produce co-occluded genotype mixtures, SfCOL-(A+C), -(A+D) and -(A+F), the methodology described previously was followed; after mixing the OBs of the different genotypes, ODVs were released by alkali disruption with a dissociation buffer (1 vol. OB: 1 vol. Na_2_CO_3_ 0.5M: 5 vol. H_2_O). Undissolved OBs and other particulate materials were pelleted by low speed centrifugation at 2,700 x g, 5 min. ODV suspension was then injected (5 µl/larva) into *S. frugiperda* fourth instars that were individually maintained on semisynthetic diet until death. OBs containing co-occluded genotypes were recovered from virus killed larvae and used to perform bioassays. Co-occlusion was not checked, as previous studies on SfMNPV have demonstrated that this methodology can generate co-occlusion of multiple genotypes within the same OB and co-envelopment within the same ODV [20].

### Insect bioassays

The mean lethal concentration (LC_50_), mean time to death (MTD) and total OB production (OBs/larva) of SfCOL-wt and each of the cloned genotypes were estimated by peroral bioassay following the droplet feeding technique [21] using the first insect colony. Bioassays were also performed using experimental mixtures of OBs, SfCOL-(A)+(C), SfCOL-(A)+(D) and SfCOL-(A)+(F), and co-occluded mixtures, SfCOL-(A+C), -(A+D) and -(A+F), comprising equal proportions of each genotype using the second colony established one year later. Second-instars of both S*. frugiperda* colonies were starved for 8 to 12 h at 26°C and then allowed to drink from an aqueous suspension containing 10% (wt/vol) sucrose, 0.001% (wt/vol) Fluorella blue, and OBs at one of the following five concentrations: 1.2x10^6^, 2.4x10^5^, 4.8x10^4^, 9.6x10^3^ and 1.92x10^3^ OBs/ml. This range of concentrations was previously estimated to result in 95 to 5% mortality [10]. Larvae that ingested the suspension within 10 min. were transferred to individual plastic cups with semisynthetic diet. Bioassays with 24 larvae per virus concentration and 24 larvae as control were performed three times. Larvae were reared at 25 °C and mortality was recorded every 12 h until the insects had either died or pupated. Virus induced mortality was subjected to logit regression using the Generalized Linear Interactive Modeling (GLIM) program [22].

Time mortality data were subjected to Weibull survival analysis using the GLIM program [22] and OB concentrations used for time mortality assays were those that resulted in ~90% larval mortality in the previous bioassays. For SfCOL-wt and the individual genotypes (SfCOL-A to -J) the 90% lethal concentrations were 1.4x10^7^, 1.1x10^6^, 9.3x10^6^, 7.0x10^6^, 2.7x10^7^, 3.7x10^6^, 1.5x10^7^, 1.9x10^6^, 5.8x10^7^, 3.9x10^7^ and 7.2x10^7^ OBs/ml, respectively. For experimental mixtures of occlusion bodies the 90% lethal concentrations were 1.4x10^6^, 2.7x10^6^ and 2.4x10^6^ OBs/ml for SfCOL-(A)+(C), SfCOL-(A)+(D) and SfCOL-(A)+(F), respectively. Finally, for co-occluded mixtures SfCOL-(A+C), SfCOL-(A+D) and SfCOL-(A+F) the 90% lethal concentrations were 3.7x10^6^, 1.3x10^6^ and 6.9x10^6^ OBs/ml, respectively. Groups of 24 second instars were infected using the droplet feeding method [20] and mortality was checked at intervals of 8 h until death. The assay was performed on three occasions. Groups of 24 control larvae were treated identically but did not feed on OB suspensions.

The production of OBs in insects infected by SfCOL-wt, single genotypes and experimental OB mixtures and co-occluded mixtures were determined in groups of 24 overnight-starved second instars inoculated with the LC_90_ used in the speed of kill assay and reared on semisynthetic diet at 25 °C until death. The whole assay was performed on three occasions. All the larvae that died from polyhedrosis disease (at least 20 for each virus treatment per replicate, a total of ~60 larvae per virus treatment) were individually collected and stored at -20 °C. For OB counting, each larva was individually homogenized in 100 µl of distilled water and counted in triplicate in a Neubauer hemocytometer. The results were analyzed by Kruskal-Wallis and Mann-Whitney nonparametric statistics using the SPSS program (SPSS version 10.0). Critical probability values were subjected to false discovery rate adjustment for multiple pairwise comparisons [23].

## Results

### Identification of genotypic variants

**Figure 1:**
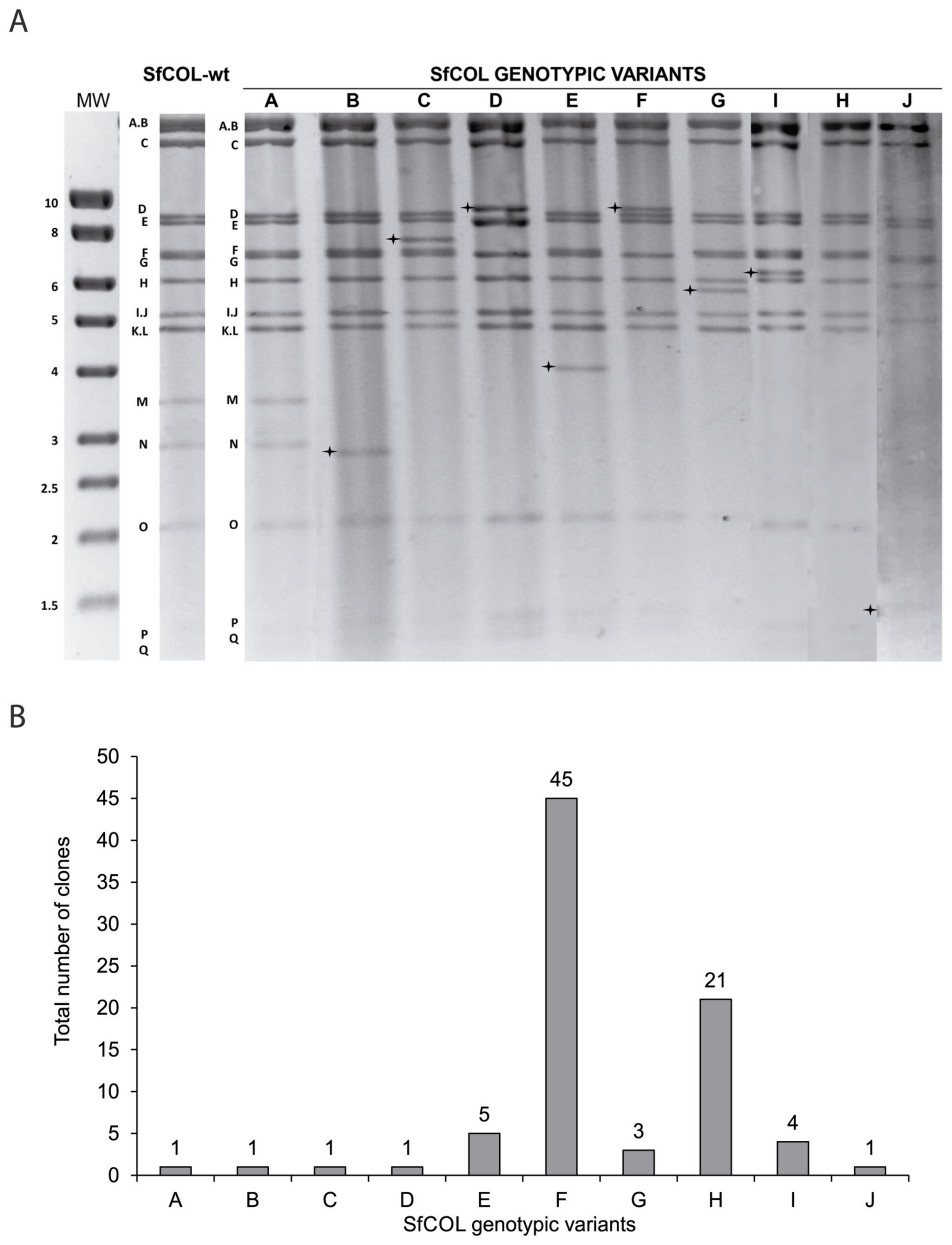
Restriction profiles and frequency of SfCOL genotypic variants. A. REN analysis of SfCOL-wt and genotypic variants DNAs digested with *Pst*I following electrophoresis in 1% agarose gel. All DNA fragments of SfCOL-wt and SfCOL-A are marked with a letter corresponding to their sizes. The cross marks the polymorphic fragments of each variant. The first lane indicates the molecular weight (MW) (1Kb DNA marker, Stratagene). B. Number of clones obtained for each of the genotypes (total number of clones 83).

**Figure 2:**
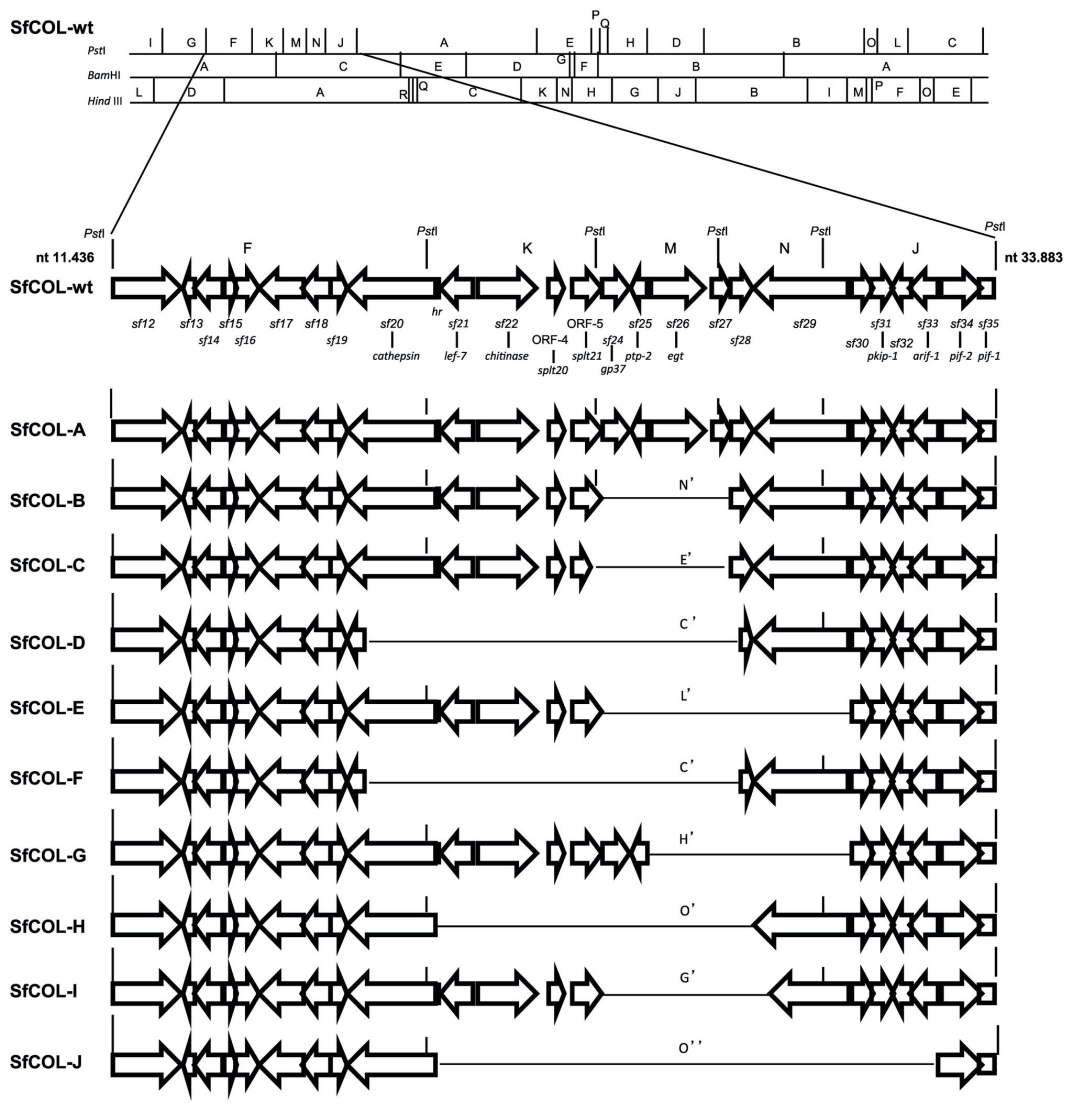
Physical maps of the variable regions of SfCOL genotypic variants. Schematic representation of the gene order of SfCOL genotypic variable region, including *Pst*I-F, -K, -M, -N, and -J fragments. The arrows represent the ORFs and point to their directions of transcription. Gene name and homologous ORFs in SfMNPV genome are indicated below the SfCOL-wt genome.

Out of 248 plaque picks, just 83 plaque picks were successfully amplified and caused fatal infection following injection in insect larvae. Ten different genotypes (named SfCOL-A to -J) were identified by analysis of plaques using *Pst*I, *Bam*HI and *Hin*dIII endonucleases (Figure 1A). The SfCOL-F genotype was the most frequently isolated genotype, which was present in 45 of amplified plaques (representing 54% of the clones), followed by SfCOL-H (N=21, 25%), SfCOL-E (N=5, 6%), SfCOL-I (N=4, 4.8%) and -G (N=3, 3.6%). The remaining genotypes appeared in only a single amplified plaque in each case (Figure 1B). All of these variants could be differentiated using the *Pst*I enzyme (Figure 1A). SfCOL-A genotype with the complete genome showed a *Pst*I restriction profile indistinguishable to that of SfCOL-wt, suggesting its high frequency in the population. While, all the others genotypes lacked specific fragments present in SfCOL-A. The *Pst*I-F fragment was absent in the SfCOL-D and -F genotypes, which differed only in the *Pst*I-D fragment that was absent in the SfCOL-D profile. The *Pst*I-K fragment was absent in five of nine deletion variants (SfCOL-C, -D, -F, -H and -J), and all genotypes (except SfCOL-A) lacked the *Pst*I-M and *Pst*I-N fragments. The *Pst*I-J fragment was absent in SfCOL-E, -G, -I and -J variants. Finally, *Pst*I-O and -L fragments were absent in SfCOL-J. In all cases missing restriction fragments were associated with the presence of additional fragments. One fragment of 9.1 Kb was shared by the SfCOL-D and -F variants. Characteristic bands were observed for unique genotypes, being of 6.3, 5.8, 4.2, 2.7, 1.9 and 1.3 Kb in SfCOL-I, -G, -E, -B, -H and -J, respectively (Figures 1A and 2, Table S2). No submolar bands were observed in these genotypes and the restriction profiles remained invariant for at least two passages in insects.

Genotype restriction fragment sizes were estimated by comparison with SfCOL-wt fragments [10] and the SfMNPV-B completely sequenced genome [18]. The SfCOL-A genome was estimated to be 133.9 kb in length, while the remaining variants were estimated between 112.0 Kb and 130.1 Kb (Table S2).

### Mapping of genotypic variants

The physical maps of the genotypes were constructed based on the physical maps of SfCOL-wt [10] (Figure 2). Genotypic variability was mainly located in a single region of the SfMNPV-B genome, between nt 11,436 and 33,883 [18], involving *Pst*I-F, -K, -M, -N and -J fragments which included 23 complete ORFs and two partial ORFs (*sf12* to *sf35*). In this region, SfCOL-J had the largest deletion of 21.8 Kb when compared with SfCOL-A, followed by SfCOL-D, -H, -F, -E, -I and -G with deletions of 10.2, 9.4, 9.3, 7.2, 5.6 and 5.1 Kb, respectively. The smallest deletion in this region of 3.8 Kb was observed in SfCOL-B and -C genotypes. Additionally a *Pst*I recognition site was absent in the SfCOL-D variant genome at nt 87,999 in the SfMNPV-B genome, which indicates an indel or mutation point at this site. Finally, SfCOL-J had a deletion in the region between nt 112,930 and 119,810 of the SfMNPV-B genome. 

The variable ORFs within SfCOL-C, -D and -F genotypes were confirmed by sequencing of *Pst*I-C' and *Pst*I-E' fragments, whereas those found in SfCOL-B, -E, -G, -H, -I and -J genomes were determined by PCR amplification using specific primers that amplified between ORFs *sf24* and *sf29* (Table S1). The variable region (12.8 Kb) among genotypes was assembled and the variable ORFs were observed between *sf20* (*cathepsin*) and *sf29*. An extended region of variability was present in SfCOL-J, which possibly included a deletion within *sf33*, corresponding to the *arif-1* gene (Figure 2). The first ORF affected (left end) was *cathepsin* (*sf20*), which presented a partial deletion of ~60% of the gene in the SfCOL-D and -F genotypes. A region including *lef-7, chitinase*, ORF4 (similar to *splt20*) and ORF5 (similar to *splt21*) was absent in the SfCOL-D, -F, -H and -J genotypes. All deletion variants lacked *gp37*, *ptp-2*, *egt* and *sf27*, except the SfCOL-G genotype that presented *gp37* and *ptp-2*. The *sf28* ORF (nt 26,351-26,998) appeared to be present in SfCOL-A because *sf27*-*sf28* (nt 26,007-26,809) and *sf28* (nt 26,602-26,809) primers amplified in this genotype. In addition, sequencing of the specific fragments of SfCOL-C, -D and -F genotypes revealed that the *sf28* ORF was present in the SfCOL-C variants, but was partially deleted in SfCOL-D and -F genotypes. In the last two genotypes, an amplification of the expected size was obtained with *sf28* primers, however these primers amplified 251 nt downstream from the start codon [18]. Finally, in SfCOL-B the amplification obtained with *sf28* primers and the size of the specific fragment (2.7 Kb), suggested the presence of this ORF. In contrast, the lack of amplification of the *sf29* ORF in SfCOL-E, -G and -J variants suggested the absence of this ORF. SfCOL-D genotype DNA was used to amplify the ends of the ORFs included in the *Pst*I-D fragment (*sf82* to *sf92*) [18]. All ORFs were amplified with identical sized products to those of SfCOL-wt, indicating the presence of these ORFs in SfCOL-D (Table S1). However, no *Pst*I recognition site was detected at nt 87,999 within the *sf92* gene, as the fragment amplified with *sf91* forward and *sf92* reverse primers of 2,829 bp did not digest with this enzyme, probably due to a point mutation, as the amplified PCR products were identical to SfCOL-wt. 

### Relative proportion of SfCOL-A genotype in the wild-type population

Sequence analysis revealed that the *egt* gene and *sf27* ORF were the only two genes absent in all deleted genotypes and were only present in the complete SfCOL-A genotype (Figure 2), which permitted the use of both genes as qPCR markers for this genotype, although the *egt* gene was selected for this purpose. qPCR analysis revealed that the SfCOL-A genotype accounted for 70.75±2.32% (mean ± SE) of the genotypes in the wild-type population.

### Biological activity of genotypic variants

The biological activities of the SfCOL genotypic variants OBs were compared with that of SfCOL-wt OBs. The OBs of all genotypes were orally infective, however SfCOL-A OBs were approximately 4.4-fold more potent (in terms of concentration mortality-metrics) than SfCOL-wt OBs. In contrast, the potency of OBs of SfCOL-B, -C, -E, -G or -I did not differ significantly from that of SfCOL-wt, and four genotypic variants (SfCOL-D, -F, -H and -J) were significantly less pathogenic than SfCOL-wt OBs (Table 1). Variants SfCOL-D, -F, -H and –J did not liquefy the infected larvae, likely due to absence of *chitinase* in all of them and/or *cathepsin* in SfCOL-D and -F.

**Table 1:** Estimated 50% lethal concentration (LC_50_), relative potency and mean time to death (MTD) values of SfCOL genotypic variant OBs in *Spodoptera frugiperda* second instars.

Virus	LC_50_ (OBs/ml)	Fiducial limits (95%)		Relative potency	*P* value	MTD (h)	Fiducial limits (95%)	
		Low	High				Low	High
SfCOL-wt	1.03x10^5^	4.40x10^4^	1.40x10^5^	1.0	-	167de	160	175
A	2.34x10^4^	1.24x10^4^	4.30x10^4^	4.4	0.001	178e	171	186
B	1.99x10^5^	1.11x10^5^	3.52x10^5^	0.5	0.102	154bcd	146	162
C	9.02x10^4^	5.05x10^4^	1.61x10^5^	1.1	0.740	151bc	144	158
D	3.31x10^5^	1.87x10^5^	5.85x10^5^	0.3	0.005	158cd	151	166
E	1.32x10^5^	7.39x10^4^	2.33x10^5^	0.8	0.538	140b	133	148
F	2.53x10^5^	1.43x10^5^	4.51x10^5^	0.4	0.025	124a	119	130
G	7.17x10^4^	3.93x10^4^	1.28x10^5^	1.4	0.357	160cd	153	168
H	2.85x10^5^	1.61x10^5^	5.04x10^5^	0.4	0.012	125a	120	131
I	1.95x10^5^	1.10x10^5^	3.45x10^5^	0.5	0.110	134ab	127	141
J	4.60x10^5^	2.58x10^5^	8.22x10^5^	0.2	<0.001	126ab	119	133

Logit regressions were fitted using GLIM program. A test for non-parallelism for all treatments was significant (Χ^2^ = 58.2, d.f. = 10, *P.*= 0.001). The SfCOL-wt, SfCOL - B, - C, - D, - F, - H, and - I genotypes presented a common slope (±SE) of 0.728 ± 0.079 whereas SfCOL-A, - E, - G and - J genotypes presented a common slope (±SE) of 0.940 ± 0.091. Relative potencies of the genotypes were calculated as the ratio of LC_50_ values relative to that of the wild-type isolate. MTD values were estimated by Weibull analysis; values labeled with different letters differed significantly (t-test, *P*<0.05).

Mean time to death values were estimated for virus concentrations that resulted in ~80% larval mortality. SfCOL-F and -H were the fastest-killing genotypic variants, followed by SfCOL-J, -I, and -E, that were significantly more virulent than SfCOL-A, -D and -G genotypic variants, that were similar to SfCOL-wt (Table 1). 

**Figure 3:**
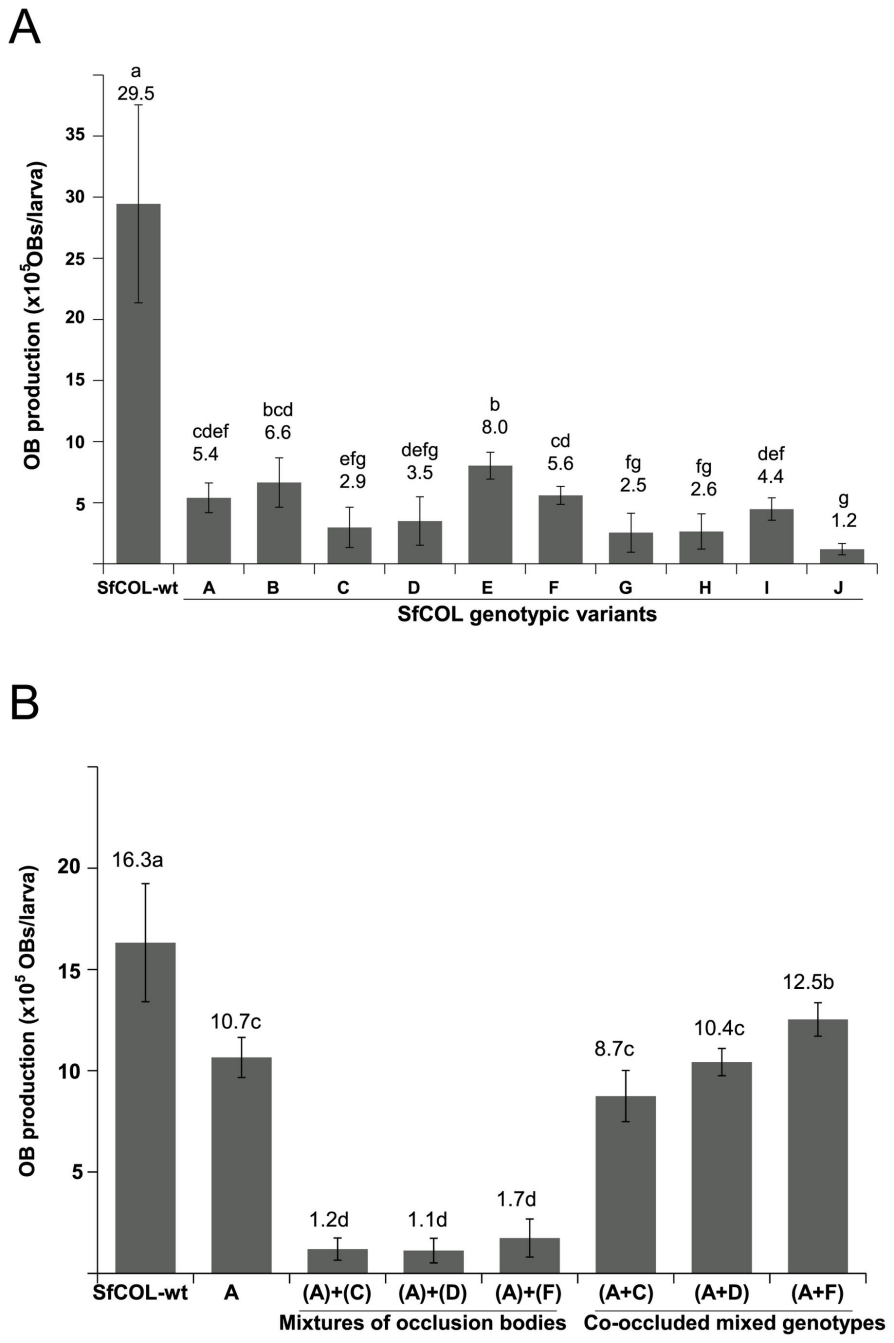
Production of occlusion bodies (OBs) in second instar *S. frugiperda*. A. Median OB production per larva of SfCOL-wt and genotypic variants in *S. frugiperda* inoculated in the second instar. B. Median OB production per larva of SfCOL-wt, SfCOL-A and OB mixtures and co-occluded genotype mixtures comprising equal proportions of SfCOL-A and -C, -D or –F genotypes. Vertical lines indicate the interquartile range. Values followed by different letters indicate significant differences (P≤0.05).

The results of OB production of the different SfCOL genotypic variants were not normally distributed and were subjected to non-parametric analysis (Figure 3A). Median OB production in insects infected by SfCOL-wt was significantly higher than that of any of the individual genotypic variants. Significant differences in OB production were also detected between the component genotypes; SfCOL-E had the highest median OB production and SfCOL-J the lowest. There was no significant relationship between speed of kill and median OB production in insects infected by these genotypes (correlation coefficient R^2^ = 0.028).

### Biological activity of genotypic mixtures

In order to study the interaction between genotypes, bioassays were performed using insects from a colony that had been recently refreshed with field-collected larvae. Experimental mixtures of OBs were prepared using the most pathogenic genotype (SfCOL-A) in equal proportions with one of the least pathogenic genotypes (SfCOL-D and -F) or a variant of intermediate potency (SfCOL-C) (Table 2). Mixtures of OBs of SfCOL-A with SfCOL-C, -D or -F OBs had potencies similar to that of SfCOL-wt OBs (Table 2). In effect, the pathogenicity of SfCOL-A genotype was reduced due to dilution by less pathogenic genotype OBs. MTD values in insects that consumed mixtures of OBs were similar to that of insects that consumed SfCOL-wt OBs or SfCOL-A OBs (Table 2). In all cases, median OB production in insects that consumed OB mixtures was approximately 10-fold lower than in insects infected by SfCOL-wt (Figure 3A), and was not significantly affected by the genotypic composition of OB mixtures. Co-occlusion of genotype A in mixtures with genotypes -C, -D or -F resulted in OBs that were of similar pathogenicity to SfCOL-wt OBs (Table 2). In contrast, the MTD of insects that consumed co-occluded mixtures of SfCOL-(A+D) or -(A+F) were significantly reduced compared to insects infected by SfCOL-(A+C), SfCOL-A alone or SfCOL-wt (Table 2). Median OB production in insects that consumed co-occluded genotypes SfCOL-(A+F) was significantly higher than that of SfCOL-(A+C) or -(A+D), both of which were similar to the median OB production value of insects infected by SfCOL-A alone (Figure 3B). No significant relationship was observed between MTD and median OB production values in insects that consumed co-occluded genotype mixtures. 

**Table 2:** Estimated LC_50_ values, relative potencies and mean time to death (MTD) values of SfCOL-wt, SfCOL-A OBs alone, mixtures of OBs, and co-occluded genotype mixtures, in similar proportions, in *Spodoptera frugiperda* second instars.

Virus	LC_50_ (OBs/ml)	Fiducial limits (95%)		Relative potency	*P* value	MTD (h)	Fiducial limits (95%)	
		Low	High				Low	High
SfCOL-wt	1.09 x10^5^	6.68x10^4^	1.76x105	1.0	-	142b	132	151
A	2.69x10^4^	1.54x10^4^	4.62x104	3.7	0.005	144b	134	154
Mixtures of occlusion bodies								
(A)+(C)	4.44x10^4^	3.22x10^4^	6.17x104	2.5	0.010	161b	147	177
(A)+(D)	9.02x10^4^	6.55x10^4^	1.25x105	1.2	0.580	160b	146	175
(A)+(F)	7.84x10^4^	5.70x10^4^	1.08x105	1.4	0.320	142b	131	154
Co-occluded mixed genotypes								
(A+C)	5.36x10^4^	3.09x10^4^	9.20x104	1.9	0.145	142b	132	151
(A+D)	7.69x10^4^	4.48x10^4^	1.33x105	1.3	0.517	115a	108	121
(A+F)	8.00x10_4_	4.26x10^4^	1.39x105	1.2	0.573	120a	112	128

Logit regressions for mixtures of occlusion bodies (OBs) were fitted using GLIM program with a common slope (±SE) of 0.850 ± 0.069. A test for non-parallelism was not significant (Χ^2^ = 7.31, df = 4, *P* = 0.12). Logit regressions for co-occluded mixed genotypes were fitted using GLIM program with a common slope (±SE) of 0.661 ± 0.083. A test for non-parallelism was not significant (Χ^2^ = 2.76, df = 4, *P* = 0.599). Relative potencies were calculated as the ratio of effective concentrations relative to that of the wild type isolate. MTD values were estimated by Weibull analysis; values followed by different letters differed significantly (t-test, P<0.05).

Finally, differences in MTD and OB production between SfCOL-wt and SfCOL-A in assays performed with the individual genotypic variants and with their mixtures, may be related to the insect colony used, as the second colony succumbed faster to SfMNPV infection and produced fewer OBs, than the first colony.

## Discussion

The composition of the genotypic variants that comprise the active ingredient can be controlled both in terms of variant selection and relative abundance for the development of baculovirus-based insecticides, and this technology can be protected by patent [24]. Given that particularly pathogenic or fast-killing traits will usually result in improved pest control and reduced crop damage following application of the bioinsecticide, these traits have generated the greatest interest during the development of these products [8]. 

In this study, the genotypic diversity of the SfCOL-wt field isolate was studied by plaque purification. The plaques produced by SfCOL genotypic variants were small, as previously described for SfNIC genotypes [7]. In addition, a low proportion of plaque picks were amplified following injection in *S. frugiperda* larvae. This could be due to the fact that plaque picks did not contain enough virus particles to kill the host, or that cell culture conditions favored the proliferation of other variants, including defective genotypes, which contain large deletions that can affect viral replication [7,25,26]. Genotypes with shorter genomes can have replication advantages over genotypes with larger genomes leading to potential over estimates of their frequencies using cell culture quantification techniques [3,7]. This was observed in the present study in which SfCOL-F was the most frequent isolated genotype, representing 54% of the clones. However, the indistinguishable restriction profiles of SfCOL-A and SfCOL-wt and the absence of visible submolar bands in SfCOL-wt [10], suggest that SfCOL-A is likely to be the dominant genotype in the natural population. The dominance of a particular genotype in a virus population has been reported in other wild-type baculoviruses, notably SeMNPV and SfMNPV isolates [5,7]. This was also confirmed by qPCR analysis of SfCOL-wt; SfCOL-A genotype accounted for 71% of the genomes amplified from the wild-type population, although this variant represented just 1.2% of the amplified clones. In theory, the restriction profile of SfCOL-A should be different from that of SfCOL-wt, as this variant comprised 71% of the genotypes in the population, however it was indistinguishable probably due to the high frequency of other genotypes with restriction profiles similar to that of SfCOL-A, such as SfCOL-H.

It is known that deletion genotypes can be generated during replication *in vitro* [25], and the difference between the frequencies of cell culture isolated genotypes and those present within the wild-type population might suggest that minority genotypes were artifacts produced during propagation of SfCOL in cell culture. However this difference has also been observed previously in the Nicaraguan isolate of SfMNPV [7,27]. Variants isolated *in vitro* at low frequency from the Nicaraguan isolate were demonstrated to be present in the wild-type population [27]. This led us to believe that SfCOL rare genotypes were not artifacts of cell culture. In this sense, SfCOL-A was selected as the reference genotype due to its high frequency in the wild-type population and because it had the largest genome.

Strikingly, the main variable region among SfCOL genotypes was similar in size and collinear with that of SfMNPV isolates from Nicaragua and Missouri [7,11]. Genotypic variation in baculoviruses is very common in natural populations and can be the result of natural intragenomic recombination in hot spot regions that contain *bro* genes, homologous regions or transposable elements [8]. Sequence analysis of the variable region in SfCOL variants revealed the presence of a single *hr*, that is identical and collinear with *hr2* reported in other SfMNPV isolates [11,18,28]. The variability around the *hr* also seems to be a common characteristic in other nucleopolyhedroviruses including SeMNPV [5], *H. armigera* NPV [29] and *Bombyx mori* NPV [30], suggesting that *hrs* are hot spots for intragenomic variation [8]. In SfMNPV, this region comprises ORFs between *sf20* to *sf29* that encode non-essential proteins with auxiliary functions, including the *egt* gene that can affect the speed of kill phenotype of these viruses [11,19]. The *egt* gene encodes an ecdysteroid UDP-glucosyltransferase that can extend the infection period allowing increased production of progeny OBs in each infected insect [31]. Using a fast-killing phenotype for pest control has been considered advantageous, since pest feeding damage on plants is reduced as larvae die earlier [32]. Nine defective SfCOL genotypic variants lacked the *egt* and *sf27* genes, however only six of them presented a faster killing phenotype compared to SfCOL-wt, suggesting that other factors are likely involved in these phenotypes.

All the SfCOL variants were orally infective as their OBs produced mortality in larvae, in contrast to that observed in SfNIC variants [7], indicating that none of them lacked the *pif* genes that are essential for peroral transmission [33]. However, differences were observed in OB pathogenicity and speed of kill; the genotypic variants with the largest deletions were significantly less pathogenic than SfCOL-wt. Similarly, variants with the largest deletions were significantly less pathogenic than the other variants present in the SfNIC population [7,19], and are therefore likely to be subject to selection for variation in transmissibility under varying biotic and abiotic conditions. *Chitinase* and *cathepsin* were deleted in the least pathogenic SfCOL variants and have been reported to be absent in several genotypes cloned from other SfMNPV isolates [11,19,34]. These genes appear to act together to facilitate the release of OBs from dead larvae with corresponding improvements in horizontal transmission [34-36]. Additionally, the genomic deletion common to the low potency variants included *lef-7* and ORFs similar to *splt20* and *splt21*, suggesting that the last two ORFs could be involved in the pathogenic characteristics of these viruses as *lef-7* is known to be involved in the replication of late genes [37]. Also, some SfCOL genotypes lacked ORF *sf29* that has been reported as a viral factor that may determine the number of ODVs occluded in each OB and thereby modulate the infectivity of OBs [38]. However, no differences were observed in OB pathogenicity between SfCOL genotypes that lacked *sf29* and SfCOL-wt in the present study.

SfCOL-A OBs were 4.4-fold more potent than SfCOL-wt OBs, indicating that the presence of other genotypes diminished the pathogenicity of the population, as observed in an isolate of SeMNPV [14]. Interactions between genotypes within baculovirus populations can have positive [12,19,39], negative [14] or neutral [40] influence on transmissibility, specifically affecting factors that influence the ability of the pathogen to infect, replicate and transmit to a new host. In contrast, all individual genotypes in the SfNIC population had lower pathogenicity than the wild-type isolate; indeed, interactions between SfNIC variants increased the transmissibility of wild-type population [7,12,19]. Characterizing interactions between genotypes is therefore crucial during the selection of genotypes or mixtures of genotypes with suitable characteristics for use in biological insecticides. Experimental mixtures of OBs and mixtures of genotypes that were co-occluded in OBs were prepared using the most pathogenic variant (SfCOL-A) and one of three genotypes with lower potency (SfCOL-C, -D or -F). Both types of mixtures decreased the pathogenicity of SfCOL-A, to values similar to that of SfCOL-wt OBs, suggesting a dilution effect of the genotypes when present in mixtures with the most pathogenic genotype SfCOL-A. Similarly, SeMNPV deletion genotypes reduced the pathogenicity of OBs when mixed with complete genotypes [14]. Following inoculation with a mixture of fast and slow killing genotypes, larvae may experience a survival time that is intermediate between the two viruses [26,41]. As such, the speed of kill of of SfCOL-(A+D) and -(A+F) mixtures was faster than SfCOL-A alone or SfCOL-wt. A positive speed of kill interaction between these genotypes was only observed when they were co-occluded for reasons that are unclear but which may be related to the prevalence of fast-killing *egt*-deleted genotypes that were transmitted more efficiently when co-occluded with the complete SfCOL-A genotype.

In conclusion, the SfCOL-wt field isolate comprises a high genotypic diversity of which SfCOL-A, the most prevalent genotype in wild-type population, was the most pathogenic and was as virulent as SfCOL-wt, in terms of speed of kill. Deletion genotypes decreased OB potency but increased OB productivity. Although within SfCOL-wt OBs the SfCOL-A was the dominant genotype, SfCOL-wt was more productive than SfCOL-A, suggesting that the other deletion genotypes presented within the SfCOL-wt may modulate increases in OB production. In this sense, it seems that SfCOL-wt is structured to maximize the likelihood of transmission. Mixtures including the most pathogenic SfCOL-A genotype and genotypes with lower pathogenicity, reduced speed of kill but also reduced OB pathogenicity which is undesirable for the development of a biological insecticide. Baculovirus based bioinsecticides are normally used for inundative applications, in which large quantities of OBs are applied for the rapid suppression of the pest. As such, OB production is usually considered to be a trait of reduced importance during bioinsecticide development, except for its involvement in virus production costs. In this respect, the most pathogenic variant was SfCOL-A which presented the most suitable characteristics as the basis for a biological insecticide to control *S. frugiperda* in Colombia. 

## Supporting Information

Table S1 **Summary of the primers used to confirm the deletions within the SfCOL genotypes and to perform qPCR.**
(DOCX)

Table S2 **Restriction fragments (Kb) generated by *Pst*I treatment of SfCOL-wt DNA and component genotypic variants.**
(DOCX)
