# Supplementary material for: Deletion Genotypes Reduce Occlusion Body Potency but Increase Occlusion Body Production in a Colombian Spodoptera frugiperda Nucleopolyhedrovirus Population
Source: PLoS One. 2013 Oct 8;8(10):e77271. doi: 10.1371/journal.pone.0077271 (PMC3792916; doi:10.1371/journal.pone.0077271)
Supplement: Table S1 — Summary of the primers used to confirm the deletions within the SfCOL genotypes and to perform qPCR. (DOCX) [file pone.0077271.s001.docx]

Table S1. Summary of the primers used to confirm the deletions within the SfCOL genotypes and to perform qPCR.

| Primer | Sequence | Nucleotide position in SfMNPV-B genome | SfCOLsize (nt) | SfCOL genotypes | | | | | | | | | | |
| --- | --- | --- | --- | --- | --- | --- | --- | --- | --- | --- | --- | --- | --- | --- |
|  |  |  |  | A | B | C | D | E | F | G | H | I | J |  |
| *sf24-sf25* | 5’-actttgtcgtcgccgctgtgaa-3’ | * | 925 | 925 | ∅ | ∅ | ∅ | ∅ | ∅ | 925 | ∅ | ∅ | ∅ |  |
|  | 5’-gcgggtatatctcgcagcgca-3’ | 23.603-23.622 |  |  |  |  |  |  |  |  |  |  |  |  |
| *egt* | 5’-acagtttggtcaagacacgccga-3’ | 24.781-24.803 | 457 | 457 | ∅ | ∅ | ∅ | ∅ | ∅ | ∅ | ∅ | ∅ | ∅ |  |
|  | 5’-gagcgcttcgacggcttcgt-3’ | 25.218-25.237 |  |  |  |  |  |  |  |  |  |  |  |  |
| *sf27-sf28* | 5’-tgttggaactccaggcggatc-3’ | 26.007-26.027 | 799 | 799 | ∅ | ∅ | ∅ | ∅ | ∅ | ∅ | ∅ | ∅ | ∅ |  |
|  | 5’-gccgtggtcgtggttggca-3’ | 26.791-26.809 |  |  |  |  |  |  |  |  |  |  |  |  |
| *sf28* | 5’-agccgatattatttgacggcttcgt-3’ | 26.602-26.628 | 206 | 206 | 206 | 206 | 206 | ∅ | 206 | ∅ | ∅ | ∅ | ∅ |  |
|  | 5’-gccgtggtcgtggttggca-3’ | 26.791-26.809 |  |  |  |  |  |  |  |  |  |  |  |  |
| *sf29* | 5’-cggatcccatgcgcatcgtcg-3’ | 27.917-27.937 | 550 | 550 | 550 | 550 | 550 | ∅ | 550 | ∅ | 550 | 550 | ∅ |  |
|  | 5’-agcgttgtttaaagaagaacgcggc-3’ | 27.390-27.411 |  |  |  |  |  |  |  |  |  |  |  |  |
| *sf82* | 5’-aacagcggcaatcggacgca-3’ | 79.177-79.196 | 329 | nt | nt | nt | 329 | nt | nt | nt | nt | nt | nt |  |
|  | 5’-gtcagttgctgttggcacacg-3’ | 79.485-79.505 |  |  |  |  |  |  |  |  |  |  |  |  |
| *sf84* | 5’-accgtttgcgcccaccacat-3’ | 80.145-80.164 | 612 | nt | nt | nt | 612 | nt | nt | nt | nt | nt | nt |  |
|  | 5’-cgcgccaacagcaaaggagc-3’ | 80.737-80.756 |  |  |  |  |  |  |  |  |  |  |  |  |
| *sf86* | 5’-tgagctcctccggcgtctcc-3’ | 82.065-82.084 | 338 | nt | nt | nt | 338 | nt | nt | nt | nt | nt | nt |  |
|  | 5’-tatcctggcgtggcgagcga-3’ | 82.383-82.402 |  |  |  |  |  |  |  |  |  |  |  |  |
| *sf87* | 5’-tgatctcgctccgccgggat-3’ | 83.415-83.434 | 471 | nt | nt | nt | 471 | nt | nt | nt | nt | nt | nt |  |
|  | 5’-taatggggcgccgaaggtgc’-3 | 82.964-82.983 |  |  |  |  |  |  |  |  |  |  |  |  |
| *sf88* | 5’-tcccggcggagcgagatcat-3’ | 83.416-83.435 | 419 | nt | nt | nt | 419 | nt | nt | nt | nt | nt | nt |  |
|  | 5’-ggtcgtcgccgatggtgctt-3’ | 83.815-83.834 |  |  |  |  |  |  |  |  |  |  |  |  |
| *sf89-sf90* | 5’-cgcgtgttccctgctctccg-3’ | 84.534-84553 | 662 | nt | nt | nt | 662 | nt | nt | nt | nt | nt | nt |  |
|  | 5’-ccttgatgctcgccgcccaa-3’ | 85.176-85.195 |  |  |  |  |  |  |  |  |  |  |  |  |
| *sf91* | 5’-gagcgctgcctacgtgcgat-3’ | 86.785-86.804 | 612 | nt | nt | nt | 612 | nt | nt | nt | nt | nt | nt |  |
|  | 5’-acgcgcagagaacacccagc-3’ | 87.377-87.396 |  |  |  |  |  |  |  |  |  |  |  |  |
| *sf92* | 5’-tgcacagtgaacgcatcaacatca-3’ | 88.273-88.296 | 1.342 | nt | nt | nt | 1.342 | nt | nt | nt | nt | nt | nt |  |
|  | 5’-gagagcactttgccgccggt-3’ | 89.595-89.614 |  |  |  |  |  |  |  |  |  |  |  |  |
| qPCR *DNA polymerase* | 5’-caacgtcgacaatcaaatgg-3’ | 89.480-89.499 | 109 | 109 | 109 | 109 | 109 | 109 | 109 | 109 | 109 | 109 | 109 |  |
|  | 5’-cgcatctctgtttctggtga-3’ | 89.570-89.589 |  |  |  |  |  |  |  |  |  |  |  |  |
| qPCR *egt* | 5’-ggtcgtcggttgtgaaatcg-3’ | 24.102-24.121 | 100 | 100 | ∅ | ∅ | ∅ | ∅ | ∅ | ∅ | ∅ | ∅ | ∅ |  |
|  | 5’-tcgaccagtgcttgaatgta-3’ | 24.182-24.201 |  |  |  |  |  |  |  |  |  |  |  |  |

The sizes of the PCR fragments are indicated according to SfCOL (*sf24* to *sf29*) and SfMNPV-B (*sf82* to *sf92* and qPCRs) sequence sizes [10,18].

nt indicates not tested. ∅ symbol represents absence of amplification and * indicates no homologs present in the SfMNPV-B genome.
