# Supplementary material for: Deletion Genotypes Reduce Occlusion Body Potency but Increase Occlusion Body Production in a Colombian Spodoptera frugiperda Nucleopolyhedrovirus Population
Source: PLoS One. 2013 Oct 8;8(10):e77271. doi: 10.1371/journal.pone.0077271 (PMC3792916; doi:10.1371/journal.pone.0077271)
Supplement: Table S2 — Restriction fragments (Kb) generated by PstI treatment of SfCOL-wt DNA and component genotypic variants. (DOCX) [file pone.0077271.s002.docx]

Table S2. Restriction fragments (Kb) generated by *Pst*I treatment of SfCOL-wt DNA and component genotypic variants.

| Fragment | SfCOL-wt | Genotypic variants | | | | | | | | | |
| --- | --- | --- | --- | --- | --- | --- | --- | --- | --- | --- | --- |
|  |  | A | B | C | D | E | F | G | H | I | J |
| A | 28.084 | 28.084 | 28.084 | 28.084 | 28.084 | 28.084 | 28.084 | 28.084 | 28.084 | 28.084 | 28.084 |
| B | 24.926 | 24.926 | 24.926 | 24.926 | 32.926 | 24.926 | 24.926 | 24.926 | 24.926 | 24.926 | 24.926 |
| C | 12.465 | 12.465 | 12.465 | 12.465 | 12.465 | 12.465 | 12.465 | 12.465 | 12.465 | 12.465 | 12.465 |
| Additional C' | 0 | 0 | 0 | 0 | 9.133 | 0 | 9.133 | 0 | 0 | 0 | 0 |
| D | 8.864 | 8.864 | 8.864 | 8.864 | 0 | 8.864 | 8.864 | 8.864 | 8.864 | 8.864 | 8.864 |
| E | 8.481 | 8.481 | 8.481 | 8.481 | 8.481 | 8.481 | 8.481 | 8.481 | 8.481 | 8.481 | 8.481 |
| Additional E' | 0 | 0 | 0 | 7.548 | 0 | 0 | 0 | 0 | 0 | 0 | 0 |
| F | 7.079 | 7.079 | 7.079 | 7.079 | 0 | 7.079 | 0 | 7.079 | 7.079 | 7.079 | 7.079 |
| G | 6.932 | 6.932 | 6.932 | 6.932 | 6.932 | 6.932 | 6.932 | 6.932 | 6.932 | 6.932 | 6.932 |
| Additional G' | 0 | 0 | 0 | 0 | 0 | 0 | 0 | 0 | 0 | 6.300 | 0 |
| H | 6.124 | 6.124 | 6.124 | 6.124 | 6.124 | 6.124 | 6.124 | 6.124 | 6.124 | 6.124 | 6.124 |
| Additional H' | 0 | 0 | 0 | 0 | 0 | 0 | 0 | 5.800 | 0 | 0 | 0 |
| I | 5.182 | 5.182 | 5.182 | 5.182 | 5.182 | 5.182 | 5.182 | 5.182 | 5.182 | 5.182 | 5.182 |
| J | 4.899 | 4.899 | 4.899 | 4.899 | 4.899 | 0 | 4.899 | 0 | 4.899 | 0 | 0 |
| K | 4.887 | 4.887 | 4.887 | 0 | 0 | 4.887 | 0 | 4.887 | 0 | 4887 | 0 |
| L | 4.769 | 4.769 | 4.769 | 4.769 | 4.769 | 4.769 | 4.769 | 4.769 | 4.769 | 4.769 | 0 |
| Additional L' | 0 | 0 | 0 | 0 | 0 | 4.200 | 0 | 0 | 0 | 0 | 0 |
| M | 3.575 | 3.575 | 0 | 0 | 0 | 0 | 0 | 0 | 0 | 0 | 0 |
| N | 2.953 | 2.953 | 0 | 0 | 0 | 0 | 0 | 0 | 0 | 0 | 0 |
| Additional N' | 0 | 0 | 2.700 | 0 | 0 | 0 | 0 | 0 | 0 | 0 | 0 |
| O | 2.112 | 2.112 | 2.112 | 2.112 | 2.112 | 2.112 | 2.112 | 2.112 | 2.112 | 2.112 | 0 |
| Additional O' | 0 | 0 | 0 | 0 | 0 | 0 | 0 | 0 | 1.921 | 0 |  |
| Additional O'' |  |  |  |  |  |  |  |  |  |  | 1.300 |
| P | 1.339 | 1.339 | 1.339 | 1.339 | 1.339 | 1.339 | 1.339 | 1.339 | 1.339 | 1.339 | 1.339 |
| Q | 1.228 | 1.228 | 1.228 | 1.228 | 1.228 | 1.228 | 1.228 | 1.228 | 1.228 | 1.228 | 1.228 |
| Total | 133.899 | 133.899 | 130.071 | 130.032 | 123.674 | 126.672 | 124.538 | 128.272 | 124.405 | 128.772 | 112.004 |

Additional fragments that did not appear in the SfCOL-wt restriction profiles are indicated using an apostrophe ('). The fragments sizes were confirmed by sequencing and/or by comparison with restriction fragments of the SfNIC genome.
